# Chinese herbal medicines for the treatment of depression: a systematic review and network meta-analysis

**DOI:** 10.3389/fphar.2024.1295564

**Published:** 2024-04-03

**Authors:** Chun Dang, Qinxuan Wang, Qian Li, Ying Xiong, Yaoheng Lu

**Affiliations:** ^1^ Department of Periodical Press, West China Hospital, Sichuan University, Chengdu, China; ^2^ West China School of Medicine, Sichuan University, Chengdu, China; ^3^ Department of Neurology, The Second Affiliated Hospital of Harbin Medical University, Harbin, China; ^4^ Department of General Surgery, Chengdu Integrated Traditional Chinese Medicine and Western Medicine Hospital, Chengdu, China

**Keywords:** bayesian network meta-analysis, Chinese herbal medicine, depression, treatment, antidepressant

## Abstract

**Objectives:** Amidst rising global burden of depression and the associated challenges with conventional antidepressant therapies, there is a growing interest in exploring the efficacy and safety of alternative treatments. This study uses a Bayesian network meta-analysis to rigorously evaluate the therapeutic potential of Chinese herbal medicines in the treatment of depression, focusing on their comparative efficacy and safety against standard pharmacological interventions.

**Methods:** Five databases (PubMed, Wanfang Data, EMBASE, CNKI, and the Cochrane Library) and grey literature were searched from inception to end of July 2023 to identify studies that assessed the efficacy and safety of Chinese herbal medicines in treating depression. The response rate, Hamilton Depression Scale (HAMD) scores, and rates of adverse events were assessed through both direct and indirect comparisons. Data extraction and risk of bias assessment were meticulously performed. Statistical analysis used Markov chain Monte Carlo methods, with effect size estimates provided as odd ratios and their 95% confidence intervals.

**Results:** A total of 198 RCTs involving 8,923 patients were analyzed, assessing 17 Chinese herbal medicines. Surface Under the Cumulative Ranking results indicated that the top three treatments with the best response rate were possibly *Guipiwan*, *Ease Pill*, and *Chaihu Jia Longgu Muli Decoction*; the top three treatments on the reduction of HAMD scores were *Chai Hu Shu Gan San*, *Xingnao Jieyu Decoction*, and *Xiaoyao Powder*; and the top three treatments with the lowest adverse effects rates were *Xiaoyao Powder*, *Alprazolam*, and *Xingnao Jieyu Decoction*. Interestingly, commonly used synthetic drugs such as *Fluoxetine*, *Escitalopram*, *Amitriptyline*, *Sertraline*, *Flupentixol and Melitracen*, and *Venlafaxine*, not only appeared to be less effective than specific Chinese herbal medicines (*Gan Mai Da Zao Decoction*, *Chaihu Jia Longgu Muli Decoction*, *Chai Hu Shu Gan San*, *Danzhi-Xiaoyao-San*, and *Xingnao Jieyu Decoction*), but they were also related to substantially higher risk of adverse events.

**Conclusion:** Our findings elucidate the promising therapeutic potential of Chinese herbal medicines as viable alternatives in the treatment of depression, with certain herbs demonstrating enhanced efficacy and safety profiles. The outcomes of this study advocate for the integration of these alternative modalities into contemporary depression management paradigms. However, it underscores the necessity for larger, methodologically robust trials to further validate and refine these preliminary findings.

**Systematic review registration:** https://www.crd.york.ac.uk/PROSPERO/, identifier CRD42023452109.

## Introduction

Depression is a pervasive mental disorder that causes people to experience anhedonia (Monroe and Harkness, 2022). Depression symptoms include sadness, cognitive difficulties, which reduce patients’ quality of life and social functioning (Bosc, 2000). Depression impacts approximately 3.8% of the global population. Its prevalence is notably higher in the adult demographic, affecting about 5% of this group. According to the World Health Organization, an estimated 280 million adults across the globe are afflicted with this condition (Freitas et al., 2023).

In the pharmacotherapeutic management of depressive disorders, a diverse array of antidepressant classes is employed. These include Tricyclic Antidepressants, Selective Serotonin Reuptake Inhibitors (SSRIs), Monoamine Oxidase Inhibitors, Serotonin and Noradrenaline Reuptake Inhibitors (SNRIs), Noradrenaline Reuptake Inhibitors, and Noradrenaline and Dopamine Reuptake Inhibitors. These pharmacological agents primarily function by inhibiting the transporters implicated in the reuptake of monoamines, a mechanism crucial in the modulation of mood and affective states (Dobrek and Głowacka, 2023). Additionally, several other compounds exhibit antidepressant properties. For instance, *agomelatine* acts as an MT1 and MT2 melatonin receptor agonist and a serotonin 5HT2 receptor antagonist, while *mirtazapine* is known to antagonize adrenergic alpha2-autoreceptors, alpha2-heteroreceptors, as well as 5-HT2 and 5-HT3 receptors. More recent developments in antidepressant pharmacotherapy include agents such as desvenlafaxine, vortioxetine, and vilazodone (Fournier et al., 2010; Faquih et al., 2019).

The therapeutic efficacy of antidepressants demonstrates considerable variability across the patient population. SSRIs and SNRIs are frequently prioritized as first-line treatments, owing to their favorable safety profiles and high tolerability. Empirical studies indicate that approximately 60%–70% of individuals diagnosed with depression experience a notable improvement in symptoms following their initial course of antidepressant therapy. Symptom amelioration can often be observed within a span of several weeks. However, there remains a substantial proportion, estimated at 30%–40%, who may not exhibit an adequate response to their first prescribed medication. This subset of patients may necessitate alterations in their pharmacological regimen or the incorporation of adjunctive therapeutic approaches (Irfan, 2024).

In addition, there are numerous adverse effects are caused by modern pharmacological drugs. The adverse effects of selective SSRIs include QT prolongation, serotonin syndrome, insomnia, rashes, and hyponatremia, whereas long-term use may lead to sexual dysfunction and weight gain (Goethe et al., 2007; Nachimuthu et al., 2012). Additionally, Monoamine Oxidase Inhibitors and Serotonin Reuptake Inhibitors are associated with potentially serious reactions such as hypertensive crisis, and increased risk of suicidal ideation (Sathyanarayana Rao and Yeragani, 2009; Nobile et al., 2019; Mrozek et al., 2023). Furthermore, overdoses of tricyclic antidepressants can precipitate severe cardiac complications, including sudden heart attack, tachycardia, and ventricular fibrillation (Scala et al., 2023; Yildiz et al., 2023).

In recent years, herbal medicines are gaining interests and recognitions (Saxena et al., 2023). Numerous Chinese herbal medicines have been investigated for their potential antidepressant effects (Garg et al., 2023). Various Chinese herbal medicines have been reported to have excellent antidepressant effects compared with current synthetic pharmaceuticals, such as *Morinda Officinalis Oligosaccharide Capsule*, *Guipiwan, Jieyu Decoction*, *Shugan Jieyu Capsule*, *Wuling Capsule*, *Ease Pill*, *Yangxue Qingnao Granule*, *Yueju Pill, Buyang Huanwu Decoction*, *Chaihu Jia Longgu Muli Decoction*, *Chai Hu Shu Gan San*, *Danzhi-Xiaoyao-San*, *Gan Mai Da Zao Decoction*, *Huoxue Soup Decoction*, *Wendan Anshen Decoction*, *Xiaoyao Powder*, and *Xingnao Jieyu Decoction* (Holden, 1987; Yeung et al., 2014a; Peng et al., 2014; Zhang et al., 2014; Feng et al., 2016; Kwon et al., 2018; Zhen et al., 2022).

The pharmacodynamic mechanisms on herbal medicines in treatment of psychiatric disorders are multifaceted. Primarily, these mechanisms encompass the modulation of neuronal communication. This is achieved through the binding of specific plant-derived metabolites to neurotransmitter and neuromodulator receptors (Sarris et al., 2011). Additionally, these herbal compounds can influence neurotransmitter synthesis and overall neurological function (Sarris, 2007). Beyond these neural interactions, herbal medicines may exert their therapeutic effects by either stimulating or sedating central nervous system activity. They also play a role in regulating and supporting the healthy functioning of the endocrine system (Kumar, 2006).

Previous published studies have only compared single Chinese herb medicine, without comparisons of multiple Chinese herb medicines. Therefore, the efficacy, tolerability, or safety is not possible to ascertain on various Chinese herb medicines. In this study, we chose common Chinese herbal medicines for depression treatment. This study rigorously evaluates specific aspects on efficacy (as measured by Hamilton Rating Scale for Depression (HAMD) score and response rate) and safety (adverse effects rate) in the context of therapeutic approaches for depression. The HAMD score is the foremost clinician-rated scale used for assessing depression severity in patients diagnosed with depressive disorders (Carrozzino et al., 2020). The response rate, defined as a reduction of ≥50% in HAMD scores at the study endpoint, is a validated and commonly employed measure of depression severity (McIntyre et al., 2005). Adverse effects rate, quantifying the proportion of patients experiencing at least one adverse event relative to the total number of patients in the intervention or control group, is a widely accepted metric for evaluating safety (Weibel et al., 2020; Dean et al., 2021; De Crescenzo et al., 2022).

Thus, this Bayesian network meta-analysis aims to synthesize and assess the existing available evidence for the efficacy and safety of various Chinese herbal medicines for the treatment of depression.

## Methods

This network meta-analysis was registered in PROSPERO with accession number CRD42023452109. The protocol followed the Preferred Reporting Items for Systematic Reviews and Meta-analyses Protocol (Moher et al., 2015). The time of registration occurred was 17 May 2023. There are not any modifications about the Preferred Reporting Items for Systematic Reviews and Meta-analyses Protocol during the study. The researchable question was performed using the PICOS (Population, Intervention, Comparison, Outcome, Study design) format. Population: patients with depression. Intervention: participants received Chinese herbal medicines. Comparison: participants received modern pharmacological antidepressants, placebo, or no-treatment. Outcome: HAMD scores, the response rate, and the incidence of drug-related adverse reactions. Study design: randomized controlled trials (RCTs).

### Data searches

A systematic literature search for articles was performed in PubMed, Wanfang Data, EMBASE, CNKI, and the Cochrane Library. Grey literature was also searched. Articles were searched in English or Chinese from inception through the end of July 2023 for studies that assessed the efficacy and safety of Chinese medicines with depression. The detailed search strategy and search terms are shown in Supplementary Appendix S1.

### Study selection

Two review authors (Chun Dang and Yaoheng Lu) independently screened the titles and abstracts, and differences were resolved through discussion and consensus agreement. Studies which potentially fulfilled the inclusion and exclusion criteria were identified, then full-text reviews were performed.

### Inclusion criteria

The inclusion criteria were as follows: (1) Adult patients (≥18 years) with depressive symptoms were eligible. Depression was defined by the standardized diagnostic manuals (Blatch Armon, et al., 2023), such as the Diagnosis and Statistical Manual of Mental Disorders, Fourth Edition (DSM-IV) or later versions (Hasin et al., 2006), the International Classification of Diseases, 10th Edition (ICD-10) (Herrmann et al., 1998), the Chinese Classification of Mental Disorders, Third Edition (CCMD-Ⅲ) or later versions. (2) The intervention group received common Chinese herbal medicines, while the control group received current synthetic pharmaceuticals, placebo, or no-treatment. All forms of Chinese herbal medicines (i.e., decoctions, formula, capsules, pills, and powders) were included. Current synthetic pharmaceuticals (i.e., *Fluoxetine*, *Escitalopram*, *Amitriptyline*, *Maprotiline*, *Venlafaxine*, *Paroxetine*, *Venlafaxine*) were included. Participants who were only assigned one drug without the combination of different antidepressants or non-pharmacology treatments (i.e., cognitive-behavioral therapy, psychotherapy). (3) Outcome included HAMD scores, the clinical response rate, and the incidence of drug-related adverse reactions. (4) Only RCTs were included.

### Exclusion criteria

The exclusion criteria were as follows: (1) Treatment groups using combinations of other depression drugs; (2) Studies with missing data about HAMD scores, or the total clinical response rate. (3) Studies that were not RCTs.

### Data extraction

Two review authors (Chun Dang and Yaoheng Lu) independently extracted the data from the included studies, resolving disagreements through consensus agreement or with third-party reviewers (Qian Li). We extracted data on patients’ characteristics (age, gender, numbers, comorbidity), interventions and control group (trial groups, duration, administration), outcomes (HAMD scores, the total clinical response rate in baseline and post-treatment), and adverse events. Due to the lengthy nature of the drug names, they have been abbreviated for enhanced readability and improved visual presentation in the figures and tables.

When discrepancies were identified, the primary reviewers discussed them to reach a consensus. If the primary reviewers cannot resolve a discrepancy, a third-party reviewer is consulted. The third-party reviewer provided an independent assessment of the disputed data points. Blinding was used during the data extraction process.

### Study quality assessment

Due to the inclusion of RCTs in this study, we have used the Cochrane Collaboration’s recommended bias assessment tool, ROB 2.0, specifically designed for RCTs. ROB is widely recognized and extensively used as the predominant tool for assessing bias risk in RCTs (Higgins et al., 2011). The risk of bias was assessed in terms of the five domains: (1) Risk of bias arising from the randomization process; (2) Missing outcome data; (3) Risk of bias due to deviations from the intended interventions; (4) Risk of bias in the selection of the reported result; (4) Risk of bias in the measurement of the outcome. The risk of bias was graded as “low risk of bias”, “some concerns” and “high risk of bias”. All stages were independently performed by two authors (Chun Dang and Yaoheng Lu).

### Statistical analysis

In this study, which involves the comparison of multiple different interventions and includes a significant number of indirect comparisons, we have adopted the commonly used Markov chain Monte Carlo method (MCMC). This approach utilizes a random effects generalized linear model for Bayesian network meta-analysis (Jansen et al., 2008). The nma. fit () function is adept at performing model fitting and identifying potential outliers. The lever plots and Deviance Information Criterion (DIC) values generated by this function are instrumental in determining the most suitable effect model. The lever diagram illustrates the comparison between leverage_ik (leverage for test i in arm k) and Bayesian deviation residuals for all *I* tests across each of the *K* arms. This diagram is particularly useful for highlighting potential outliers in model fitting. Specifically, if a data point falls outside the purple arc, it may indicate poor model fitting. We used odd ratios or their logarithms as the effect index of counting data and their 95% confidence intervals (*CI*) as limits. We use mean difference as the statistical effect size for continuous variables, and OR for binary variables, based on the type of outcome data. When the odds ratio (*OR*) value did not contain 1 at the 95% *CI*, the difference was considered statistically significant. Statistical heterogeneity was assessed using the *I* ^*2*^ statistic (Chen and Benedetti, 2017). The *I* ^*2*^ statistic for assessing statistical heterogeneity, serves as a method to measure the degree of variance among multiple study effects. It specifically quantifies the percentage of total variation that is attributable to differences between studies, rather than to sampling error. The categorization of heterogeneity via the *I* ^*2*^ statistic is as follows: *I* ^*2*^ of 0 indicates that the variation among studies is solely due to sampling error; *I* ^*2*^ between 0.25 and 0.5 suggests moderate heterogeneity; and *I* ^*2*^ greater than 0.5 is indicative of high heterogeneity. Some scholars argue that the *I* ^*2*^ statistic, by applying a degrees-of-freedom adjustment, mitigates the impact of the number of included studies on the *Q* value, ensuring that its magnitude does not fluctuate with the number of studies and thus making the heterogeneity test results more robust and reliable (Higgins et al., 2003). The magnitude of publication bias is assessed by examining the distribution of individual study points within a funnel plot. If the points are symmetrically distributed on either side of the plot, it suggests a lower likelihood of publication bias. The convergence of the model was performed using the Gelman-Rubin method combined with a density plot and tractory plot (Brooks and AJJCGS, 1998). A network meta-analysis was performed for each collected outcome of studies. For different outcomes, we summarized the current evidence by drawing three network graphs. The effectiveness, and safety of different drugs in the treatment of depression were ranked based on the Surface Under the Cumulative Ranking (SUCRA) curve (Salanti et al., 2011). Pairwise meta-analysis will be conducted using Stata, version 17, and network meta-analysis within the Bayesian framework will be conducted by using R software, version 4.3.1 (R Foundation for Statistical Computing, Shanghai, Asia), with the package calling “gemtc 0.8–2” and “JAGS” (version 3.5.3) (Neupane et al., 2014; Shim et al., 2019). *p* < 0.05 was considered to indicate statistical significance.

## Results

### Screening results

**FIGURE 1:**
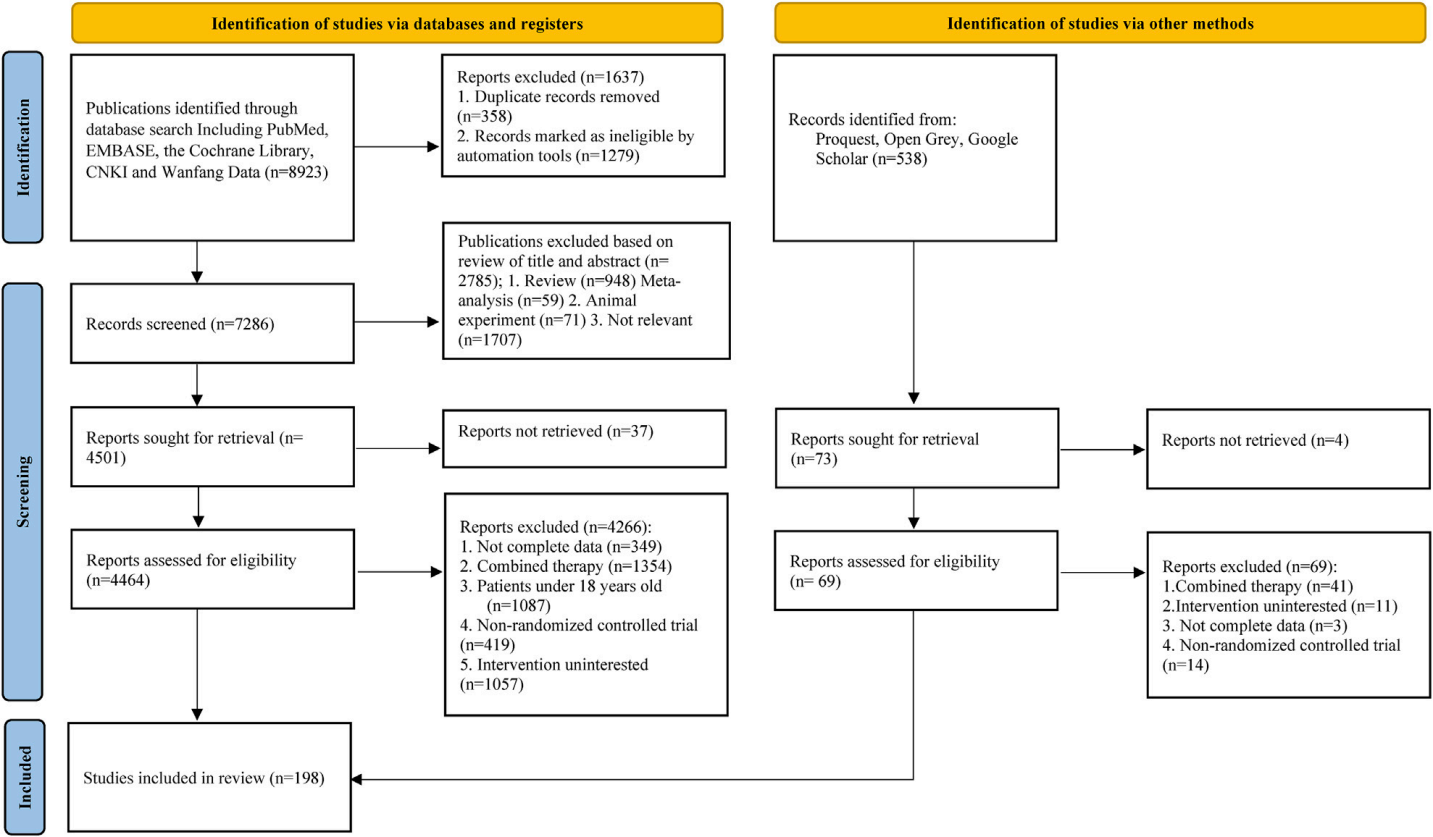
Supplementary Figure S1 Search results and study selection. This flow diagram adapted from PRISMA depicts the search results retrieved from databases and illustrates the process of literature screening.

After database retrieval, 8,923 citations were identified in five databases and 538 studies in the grey literature. Ultimately, 198 randomized control trials fulfilled the inclusion and exclusion criteria after reading the full text (Figure 1).

### Study and participant characteristics

The analysis incorporated 198 RCTs, which collectively enrolled 8,923 patients. These patients were treated with 17 Chinese herbal medicines for depression treatment. This study included six trials (646 patients) on *Morinda Officinalis Oligosaccharide Capsule*, two trials (92 patients) on *Guipiwan*, 18 trials (690 patients) on *Jieyu Decoction*, 35 trials (1,469 patients) on *Shugan Jieyu Capsule*, 15 trials (697 patients) on *Wuling Capsule*, five trials (187 patients) on *Ease Pill*, two trials (152 participants) on *Yangxue Qingnao Granule*, three trials (98 patients) on *Yueju Pill*, eight trials (326 patients) on *Buyang Huanwu Decoction*, 34 trials (1,601 patients) on *Chaihu Jia Longgu Muli Decoction*, 11 trials (391 patients) on *Chai Hu Shu Gan San*, 19 trials (834 patients) on *Danzhi-Xiaoyao-San*, 12 trials (382 patients) on *Gan Mai Da Zao Decoction*, 12 trials (644 patients) on *Huoxue Soup Decoction*, five trials (227 patients) on *Wendan Anshen Decoction*, seven trials (248 patients) on *Xiaoyao Powder*, and four trials (239 patients) on *Xingnao Jieyu Decoction*. The median follow-up period for these trials ranged from 4 weeks to 6 months. All studies were conducted in China. A detailed description of the participants is presented in Supplementary Table S1 (Cao and Zhong, 2008; Cao, 2009; Chang and Wang, 2010; Chen and Bai, 2011; Chen and Wang, 2012; Qu et al., 2012; Cao and Chi, 2017; An and Wang, 2019; Chen GXFN. and Li T., 2009; Chen KZC. and Li XX., 2009; Chen and He, 2009; Chen and Wang, 2009; Deng and Sun, 2012; Du and Yu, 2012; Chen and Dou, 2014; Chen and Wang, 2015; Ding, 2015; Chen and Ma, 2016; Cheng and Yang, 2016; Chen and Li, 2017; Chen and Zhang, 2018; Cheng and Li, 2020; Deng et al., 2022; Gong, 2010; He, 2011; Gao et al., 2012; Guo et al., 2012; Feng, 2013; Guan and Wu, 2014; Guo and Hu, 2014; Huang and Ting, 2014; Guo and Zhang, 2015; Guan et al., 2017; He et al., 2017; Guo et al., 2018; He and Wang, 2018; Hou and Wang, 2019; Guo and Li, 2020; Li and Zhang, 2004; Huang et al., 2007; Li and Li, 2008; Jing et al., 2009; Li et al., 2009; Li and Zhang, 2010; Li and Zhao, 2011; Li RGZT. and Li S., 2012; Li SHLY. and Li SS., 2012; Huang and Zhou, 2014; Li and Li, 2014; Jiao et al., 2015; Li and Gao, 2015; Li QLLJ. et al., 2016; Li SSYM. et al., 2016; Jin et al., 2017; Lai and Yi, 2017; Li and Guo, 2017; Jia et al., 2019; Li and Wang, 2019; Li et al., 2003; Li and Wang, 2004; Li, 2007; Li and Qian, 2007; Liu et al., 2007; Li and Tian, 2008; Li and Dong, 2009; Li et al., 2010; Liu and Tan, 2010; Lin et al., 2011; Liu and Zhang, 2012; Li and Luo, 2013; Li et al., 2014; Li and Wang, 2015; Liu and Wang, 2015; Liu and Wang, 2017; Li et al., 2018; Lin and Han, 2019; Liu and Wang, 2019; Li et al., 2020; Luo and Zhao, 2006; Meng et al., 2008; Mao and Li, 2010; Ran et al., 2010; Meng and Li, 2011; Qiu and Zhu, 2011; Qu et al., 2011; Mao and Li, 2016; Meng et al., 2016; Nie et al., 2016; Qin and Tang, 2016; Ma and Ye, 2017; Qi et al., 2017; Ren, 2017; Min CX and Li, 2018; Lu and Li, 2019; Luo et al., 2019; Lu and Xin, 2020; Luo and Wu, 2021; Lu and Sun, 2022; Shen and Zhao, 2004; Tao and Li, 2006; Shi and Zeng, 2008; Ta et al., 2008; Tang and Wang, 2009; Shao and Zhao, 2011; Wang and Wang, 2011; Sun and Li, 2012; Sun and Zhou, 2012; Tao and Yin, 2012; Ren and Li, 2015; Tan et al., 2015; Tao and Wang, 2015; Shao and li, 2016; Tu and Wang, 2016; Wang et al., 2016; Wang and Wang, 2018; Song and Li, 2019; Wang and Li, 2019; Wang and Shao, 2019; Wang and Ma, 2006; Wang and Luo, 2007; Wang and Wu, 2008; Wang and Shu, 2009; Wang and Ban, 2010; Wang et al., 2012; Wang et al., 2014; Wei and Huang, 2014; Wei and Lu, 2014; Wang and Liu, 2016; Wang and Yang, 2016; Wu and Zhu, 2016; Wang and Li, 2017; Wang and Liu, 2017; Wang and Dou, 2018; Wang and Zhang, 2018; Wang and Zhang, 2019; Wang and Guo, 2020; Wang and Wan, 2020; Wang and Jiang, 2021; Wu et al., 2002; Yan and Wang, 2003; Xiao and Huang, 2004; Xiao, 2006; Xu and Li, 2006; Xu et al., 2007; Xu and Wang, 2007; Yan and Li, 2008; Xun and Bai, 2010; Xu TWS. et al., 2011; Xu TSWQ. et al., 2011; Xie and Li, 2011; Wu et al., 2012; Xu and Li, 2012; Xu and Yang, 2014; Yan and Bo, 2014; Wu and Wan, 2015; Xu and Wang, 2015; Wu and Tian, 2018; Xu and Wu, 2019; Zhang and Tian, 2008; Yu and Zhao, 2010; Zhan and Wang, 2010; Zhang and Gu, 2010; Yang, 2012; Yu and Wang, 2012; Zhang and Tian, 2012; Ye and Xia, 2014; Yang and Rui, 2015; Zhang and Li, 2015; Zhang and Zhang, 2016; Yu and Zhang, 2017; Yin and Zhang, 2018; Yu and Tang, 2018; Zhang and Zhou, 2018; Yin et al., 2019; Zhang and Bai, 2019; Zhang and Gan, 2020; Zhang and Yan, 2020; Zhang and Ji, 2021; Zhou and Wang, 2005; Zhang and Zhang, 2009; Zhou and Geng, 2009; Zhou and Bao, 2010; Zhao and Hu, 2011; Zhao and Wang, 2011; Zhang and Tang, 2012; Zhao and Zhao, 2012; Zhou and Xiao, 2015; Zhao and Zhang, 2021; Du and Cai, 2005; Zhu and Yang, 2005; Dang and Chu, 2009; Liu and Ku, 2012; Zhu and Li, 2014; Guan and Wang, 2017; Liu et al., 2017; Deng and Li, 2019; Shao and Feng, 2019; Gou and Wu, 2023).

### Risk-of-bias assessment

**FIGURE 2:**
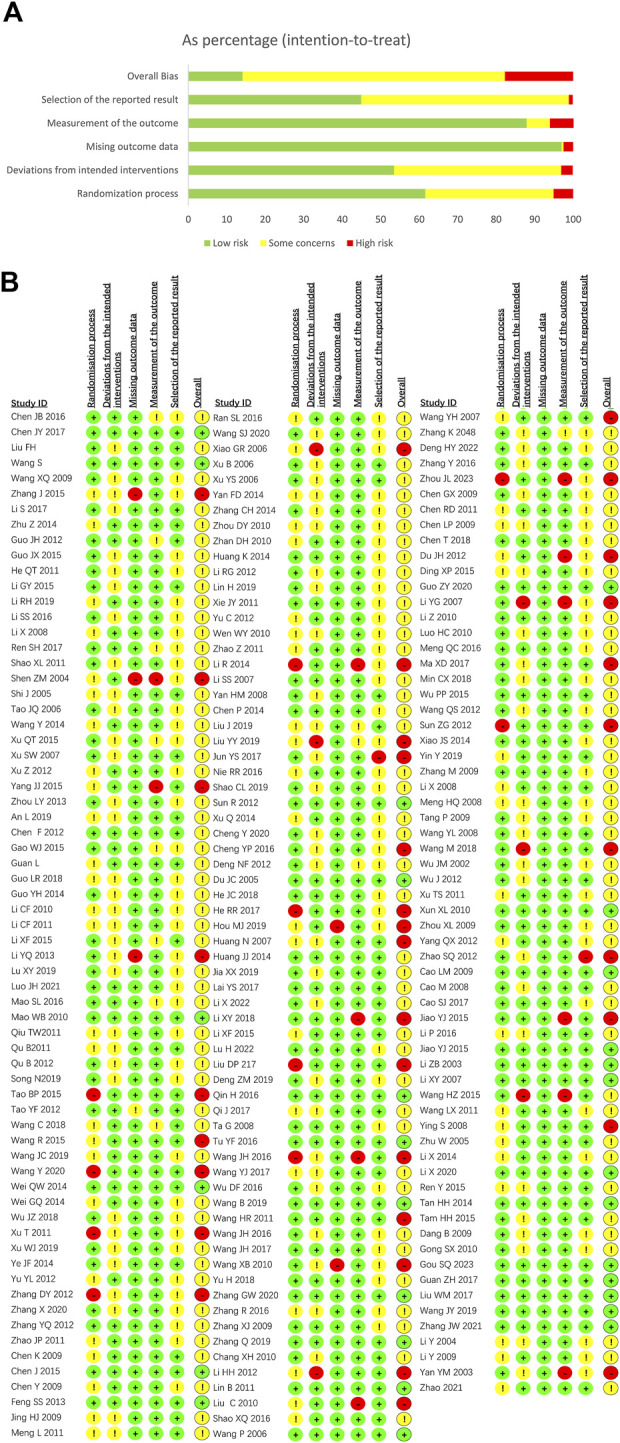
Risk assessment results for depression. **(A)** Risk of bias summary for depression; **(B)** Risk of bias graph for depression.

We comprehensively conducted a methodological quality assessment on 198 included RCTs. Based on the summary of the risk of bias, 135 studies (68.2%) were assessed as “some concerns”, 28 studies (14.1%) were rated as “low risk bias”, and 35 studies (17.7%) were classified as “high risk bias”. Overall, these factors result in an overall low-to-moderate risk of bias. The bias risk assessment results were presented in Figure 2.

### Network diagram

**FIGURE 3:**
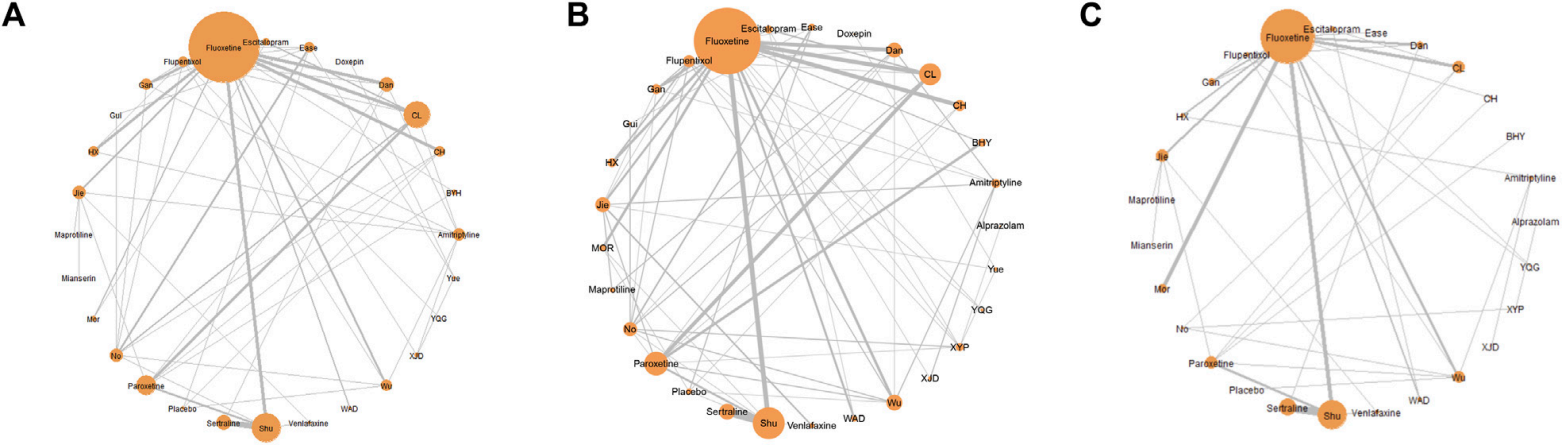
Network meta-analysis diagrams of eligible comparisons. **(A)** The response rate; **(B)** HAMD scores; **(C)** adverse effects rate. The size of nodes is proportional to the total number of participants for each group in the network. The edges represent direct comparisons of the drugs in trials. The line width is proportional to the number of trials directly compared at both ends of the nodes. Mor:Morinda Officinalis Oligosaccharide Capsule, Gui: Guipiwan, Jie: Jieyu Decoction, Shu: Shugan Jieyu Capsule, Wu: Wuling Capsule, Ease: Ease Pill, YQG: Yangxue Qingnao Granule, Yue: Yueju Pill, BYH; Buyang Huanwu Decoction, CL: Chaihu Jia Longgu Muli Decoction, CH: Chai Hu Shu Gan San, Dan: Danzhi-Xiaoyao-San, Gan: Gan Mai Da Zao Decoction, HX: Huoxue Soup Decoction, WAD: Wendan Anshen Decoction, XYP: Xiaoyao Powder, XJD: Xingnao Jieyu Decoction.

The network diagram provides a visual representation of all the studies included in the meta-analysis and their interconnections. It illustrates how each treatment is compared within the network of studies. The network diagram was outputted to describe the research network graphically. The node size is proportional to the total number of participants in each group. The line width is proportional to the number of clinical trials. When a closed loop is formed between nodes, these studies could be simultaneously compared. Among them, *Fluoxetine*, *Shugan Jieyu Capsule*, and *Chaihu Jia Longgu Muli Decoction* were more extensively studied, followed by *Paroxetine*, *Danzhi-Xiaoyao-San*, and *Jieyu Decoction*. The two groups most frequently compared were *Shugan Jieyu Capsule* and *Fluoxetine*, and *Chaihu Jia Longgu Muli Decotion* and *Fluoxetine*, respectively (Figure 3).

### Publication bias and consistency assessment

**FIGURE 4:**
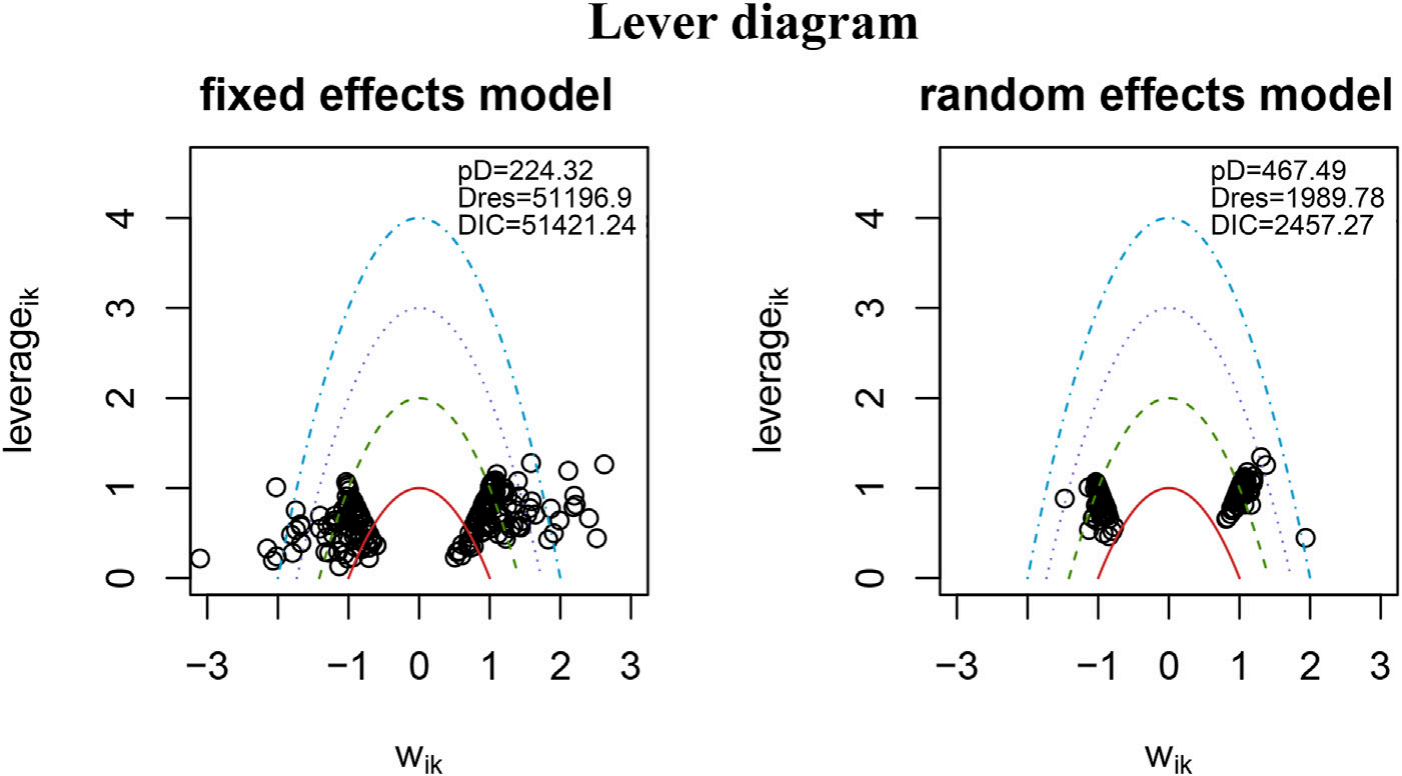
Lever diagram for depression. The lever diagram shows the comparison between leverageik and Bayesian deviation residuals of all I tests and each of the K arms.

**FIGURE 5:**
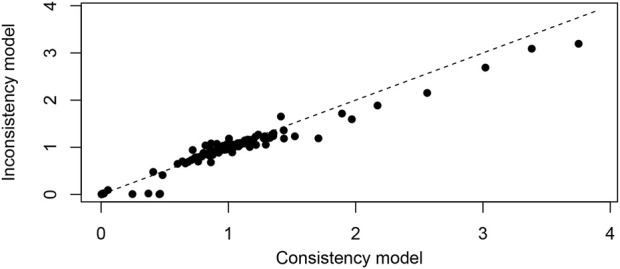
Conformance test for depression. The conformance test compares the posterior mean deviation of each data group between consistency and the ume m (b) Bias risk evaluation results displayed by including studies to judge the consistency among the included research results.

**FIGURE 6:**
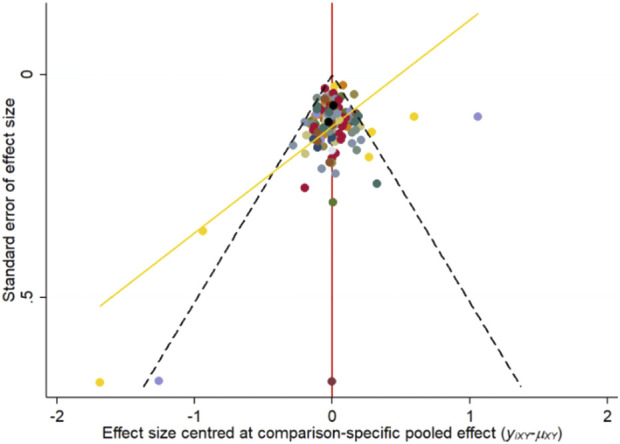
Funnel plots. The publication biases were evaluated using funnel plots.

The nma. fit () function was employed for model fitting and identification of potential outliers. The lever plots and DIC values were utilized to determine the optimal effect model (Watt and Del Giovane, 2022). Figures 4, 5 displayed the lever diagram and consistency test, respectively. Funnel plots presented a symmetrical distribution, thereby indicating limited publication bias (Figure 6).

Trajectory plots (Supplementary Figure S1A) and density plots (Supplementary Figure S1B) were used to assess the degree of convergence. The trajectory plots showed that the MCMC chain stably fluctuates and present good overlap. The density plots indicated excellent model convergence. When the curve tends to 1 and remains stable, it indicates good convergence on the Brooks-Gelman-Rubin diagnostic diagram (Supplementary Figure S2).

### Forest map

**FIGURE 7:**
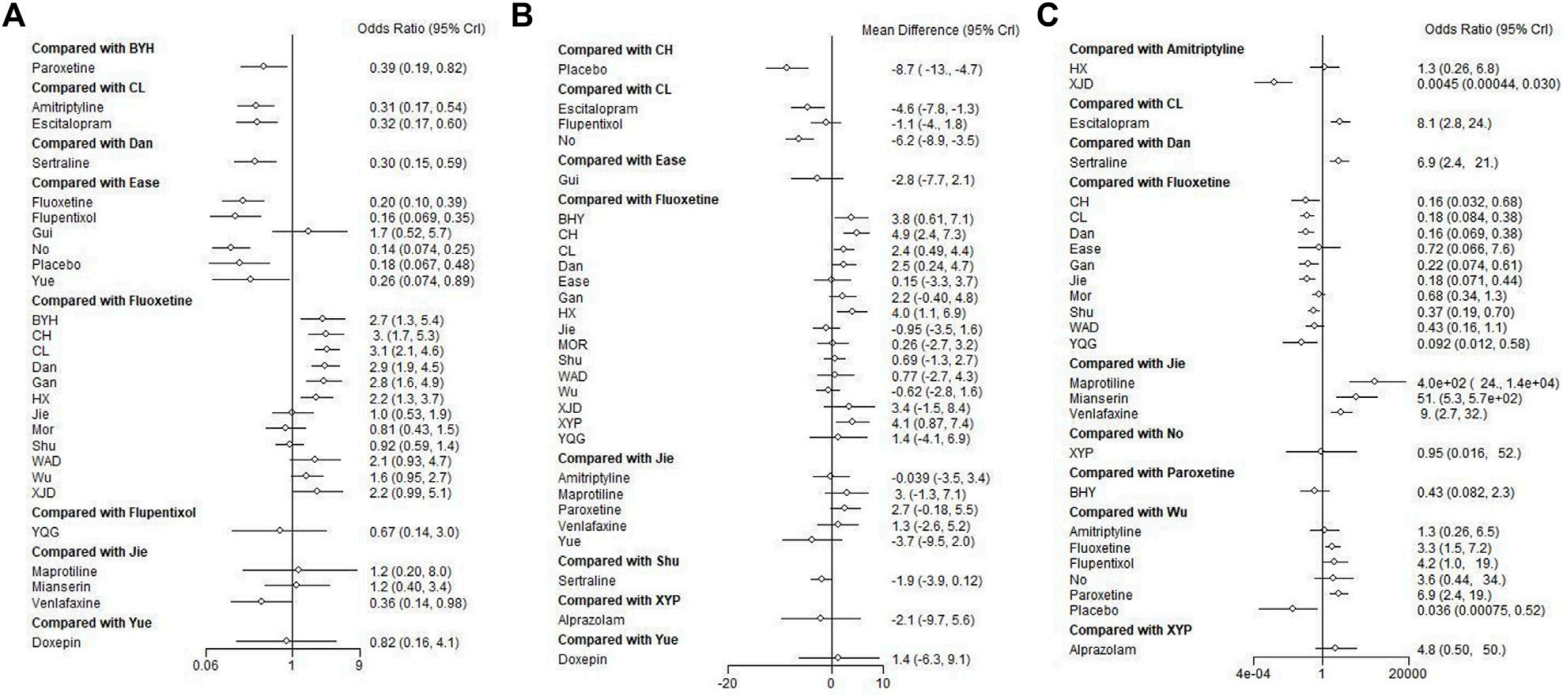
Forest plot of the network meta-analysis of all trials for efficacy and adverse effects in patients with depression. **(A)** Direct comparison of the response rate; **(B)** direct comparison of HAMD scores; **(C)** direct comparison of the adverse effects rate. Mor:Morinda Officinalis Oligosaccharide Capsule, Gui: Guipiwan, Jie: Jieyu Decoction, Shu: Shugan Jieyu Capsule, Wu: Wuling Capsule, Ease: Ease Pill, YQG: Yangxue Qingnao Granule, Yue: Yueju Pill, BYH: Buyang Huanwu Decoction, CL: Chaihu Jia Longgu Muli Decoction, CH: Chai Hu Shu Gan San, Dan: Danzhi-Xiaoyao-San, Gan: Gan Mai Da Zao Decoction, HX: Huoxue Soup Decoction, WAD: Wendan Anshen Decoction, XYP: Xiaoyao Powder, XJD: Xingnao Jieyu Decoction.

Forest map focus on these comparisons allows for a direct assessment of how alternative treatments like Chinese herbal medicines stack up against commonly used synthetic antidepressants in terms of both efficacy and safety. Forest maps compare the results of drugs, placebo, or no-treatment in various studies. The treatment efficacy of some Chinese herbal medicines was demonstrated to be generally superior to that of traditional antidepressants. Compared with those in *Fluoxetine*, *Buyang Huanwu Decoction*, *Chai Hu Shu Gan San*, *Chaihu Jia Longgu Muli Decotion*, *Danzhi-Xiaoyao-San*, *Gan Mai Da Zao Decoction*, *Huoxue Soup Decoctio*n, and *Ease Pill* groups exhibited higher response rates. In addition, interventions of *Buyang Huanwu Decoction* showed a higher response rate compared to *Paroxetine* groups. Moreover, *Amitriptyline* and *Escitalopram* were inferior to *Chaihu Jia Longgu Muli Decoction*. Additionally, *Sertraline* had a lower response rate compared to *Danzhi-Xiaoyao-San* group. Moreover, *Yueju Pill* and *Flupentixol and Melitracen*, *Venlafaxine*, with lower response rates, were comparable to the *Ease Pill* groups. *Jieyu Decoction* was demonstrated to significantly improve depressive symptoms compared to *Venlafaxine* (Figure 7A).

Compared to *Fluoxetine*, patients receiving Chinese herbal medicines, including *Buyang Huanwu Decoction*, *Chai Hu Shu Gan San*, *Chaihu Jia Longgu Muli Decoction*, *Danzhi-Xiaoyao-San*, *Huoxue Soup Decoction*, and *Xiaoyao Powder*, exhibited better efficacy in terms of HAMD scores. In particular, *Chaihu Jia Longgu Muli Decoction* presented a more promising antidepressant effect than *Escitalopram* on HAMD scores (Figure 7B).

In terms of adverse events, *Xingnao Jieyu Decoction* had a significantly lower safety risk than *Amitriptyline*. Compared with *Fluoxetine*, *Chai Hu Shu Gan San*, *Chaihu Jia Longgu Muli Decoction*, *Danzhi-Xiaoyao-San*, *Gan Mai Da Zao Decoction*, *Jieyu Decoction*, *Shugan Jieyu Capsule*, and *Yangxue Qingnao Granule* exhibited lower safety risks of adverse outcomes. Furthermore, compared with *Maprotiline*, *Jieyu Decotion* had lower safety risk. *Jieyu Decoction* had lower safety risk than *Maprotiline*, *Mianserin*, and *Venlafaxine*. *Wuling Capsule* had lower safety risk than *Fluoxetine*, *Flupentixol and Melitracen*, *Paroxetine* (Figure 7C).

### The heatmap of the ranking table

The results are presented in a heatmap format, with colors representing the magnitude of effect or ranking probability. The rows of the heatmap typically represent different treatments, while columns represent different outcome measures. Each cell in the heatmap corresponds to the intersection of the categories on the *x* and *y*-axes. The colors in a heatmap are often used to represent a gradient in continuous data. Deep red may indicate higher values, * represents statistically significant data (*p* < 0.05). For instance, in the diagram where the horizontal axis represents *Chai Hu Shu Gan San* and the vertical axis represents *Paroxetine* (**3.11***), there is a statistically significant improvement in the HAMD score for *Chai Hu Shu Gan San* compared to *Paroxetine* (*p* < 0.05).

**FIGURE 8:**
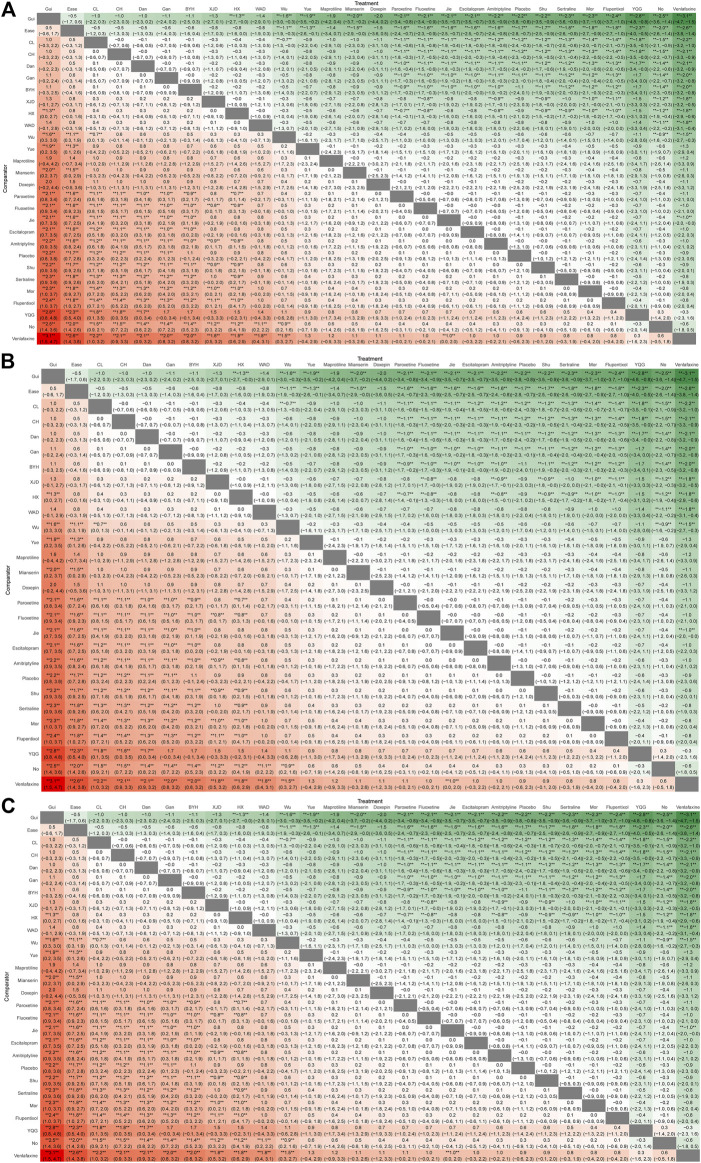
Ranking chart heatmap for depression. The heatmap of each outcome index ranking table shows comparisons of the relative effects between any pair of interventions, including the OR and 95% CI of each outcome index in all groups. **(A)** The response rate ranking chart heatmap; **(B)** HAMD scores ranking chart heatmap; **(C)** adverse effects rate ranking chart heatmap. Mor:Morinda Officinalis Oligosaccharide Capsule, Gui: Guipiwan, Jie: Jieyu Decoction, Shu: Shugan Jieyu Capsule, Wu: Wuling Capsule, Ease: Ease Pill, YQG: Yangxue Qingnao Granule, Yue: Yueju Pill, BYH: Buyang Huanwu Decoction, CL: Chaihu Jia Longgu Muli Decoction, CH: Chai Hu Shu Gan San, Dan: Danzhi-Xiaoyao-San, Gan: Gan Mai Da Zao Decoction, HX: Huoxue Soup Decoction, WAD: Wendan Anshen Decoction, XYP: Xiaoyao Powder, XJD: Xingnao Jieyu Decoction.

The heatmap of each outcome index ranking table included the 95% *CI* and *OR* of each outcome index across all groups. Interventions involving *Buyang Huanwu Decoction*, *Chai Hu Shu Gan San*, *Chaihu Jia Longgu Muli Decoction*, *Danzhi-Xiaoyao-San*, and *Ease Pill* presented more encouraging point estimates than *Escitalopram*, *Fluoxetine*, *Flupentixol and Melitracen*, *Jieyu Decoction*, *Morinda Officinalis Oligosaccharide Capsule*, *Paroxetine*, *Sertraline*, *Venlafaxine*, and *Shugan Jieyu Capsule*. Moreover, *Gan Mai Da Zao Decoction* and *Guipiwan* were statistically superior in evaluations compared to *Jieyu Decoction*, *Morinda Officinalis Oligosaccharide Capsule*, *Paroxetine*, *Sertraline*, *Venlafaxine*, and *Shugan Jieyu Capsule* (Figure 8A).

*Chai Hu Shu Gan San*, *Huoxue Soup Decoction* displayed significant effectiveness as active drugs with statistical certainty compared with *Shugan Jieyu Capsule*, *Morinda Officinalis Oligosaccharide Capsule*, *Fluoxetine*, *Wuling Capsule*, *Jieyu Decoction*, *Amitriptyline*, *Sertraline*, *Escitalopram*, *Guipiwan*, and *Yueju Pill* in terms of HAMD scores. *Buyang Huanwu Decoction* and *Xingnao Jieyu Decoction* exhibited greater efficacy than *Fluoxetine*, *Wuling Capsule*, *Jieyu Decoction*, *Amitriptyline*, *Sertraline*, *Escitalopram*, *Guipiwan*, and *Yueju Pill* in terms of HAMD scores. The efficacy of *Danzhi-Xiaoyao-San* and *Chaihu Jia Longgu Muli Decoction* was significantly greater than that of *Fluoxetine*, *Wuling Capsule*, *Sertraline*, *Escitalopram*, and *Yueju Pill* when assessed by HAMD scores (Figure 8B).

Regarding safety outcomes, treatments with a lower risk of depression-related adverse effects were *Xingnao Jieyu Decoction* and *Chai Hu Shu Gan San* (Figure 8C).

### SUCRA rankings

After preparing the data with the data. prep () function, we utilized the net. plot () function to graphically depict the research network. The net. plot () function is capable of generating a network diagram for the outcome indicators as needed. SUCRA is a numerical representation method, often presents as a percentage, which is used to summarize the comprehensive ranking of each treatment across multiple outcome indicators. This value is derived from the cumulative ranking probabilities of each treatment, that is consistent with the area under the curve. The SUCRA value is higher, the ranking of the treatment in terms of effectiveness, or safety are higher. The ranking table provides a straightforward way to compare the performance of different interventions or treatments, offers quantitative insights that are more digestible than raw statistical data. The ranking table allows for a direct comparison of the effectiveness and safety of these treatments, making it easier to understand which treatments stand out.

Treatments were ranked for the response rate, HAMD score based on SUCRA values, as illustrated in Table 1. The ranking probability histogram and cumulative probability ranking chart intuitively displayed the sorting probability of each group in Figure 8, consistent with the SUCRA rankings (Table 1).

**TABLE 1:** SUCRA rankings.

Efficacy				Safety	
The effective rate	SUCRA(%)	HAMD	SUCRA(%)	Adverse effects rate	SUCRA(%)
Gui	96.93	CH	89.96	XYP	98.23
Ease	93.76	XJD	87.40	Alprazolam	95.25
CL	83.37	XYP	84.19	XJD	91.98
CH	81.44	BHY	82.50	Placebo	91.41
Dan	80.61	HX	77.36	CH	78.51
Gan	78.58	Dan	76.00	YQG	74.75
BYH	76.71	Maprotiline	73.28	Jie	73.09
XJD	70.93	CL	68.41	CL	70.46
HX	70.55	Paroxetine	63.52	Gan	66.82
WAD	67.73	Venlafaxine	60.74	Dan	63.45
Wu	58.02	Amitriptyline	60.35	Wu	61.63
Yue	46.60	Alprazolam	59.93	Shu	53.39
Maprotiline	46.25	Jie	50.90	Amitriptyline	51.57
Mianserin	43.20	Shu	49.94	WAD	47.84
Doxepin	40.87	WAD	48.34	HX	41.46
Paroxetine	38.31	YQG	46.65	BHY	38.64
Fluoxetine	36.63	MOR	45.28	Mor	38.05
Jie	36.12	Easa	44.70	Ease	37.47
Escitalopram	35.81	Gan	43.70	Fluoxetine	28.48
Amitriptyline	33.79	Fluoxetine	39.13	Venlafaxine	28.12
Placebo	31.62	Wu	34.76	No	27.57
Shu	31.62	Flupentixol	33.32	Sertraline	26.98
Sertraline	27.86	Doxepin	31.12	Escitalopram	23.39
Mor	25.56	Sertraline	28.63	Paroxetine	20.85
Flupentixol	25.22	Gui	16.62	Mianserin	12.06
YQG	19.38	Escitalopram	16.18	Flupentixol	8.51
No	16.59	Yue	15.63	Maprotiline	0.03
Venlafaxine	5.95	Placebo	11.76		
		No	9.70		

SUCRA, surface under the cumulative ranking; Mor, Morinda Officinalis Oligosaccharide Capsule; Gui, Guipiwan; Jie, Jieyu Decoction; Shu, Shugan Jieyu Capsule; Wu, Wuling Capsule; Ease, Ease Pill; YQG, yangxue qingnao granule; Yue, Yueju Pill; BYH, buyang huanwu decoction; CL, chaihu jia longgu muli decoction; CH, chai hu shu gan san; Dan, Danzhi-Xiaoyao-San; Gan, Gan Mai Da Zao Decoction; HX, huoxue soup decoction; WAD, wendan anshen decoction; XYP, xiaoyao powder; XJD, xingnao jieyu decoction.

**FIGURE 9:**
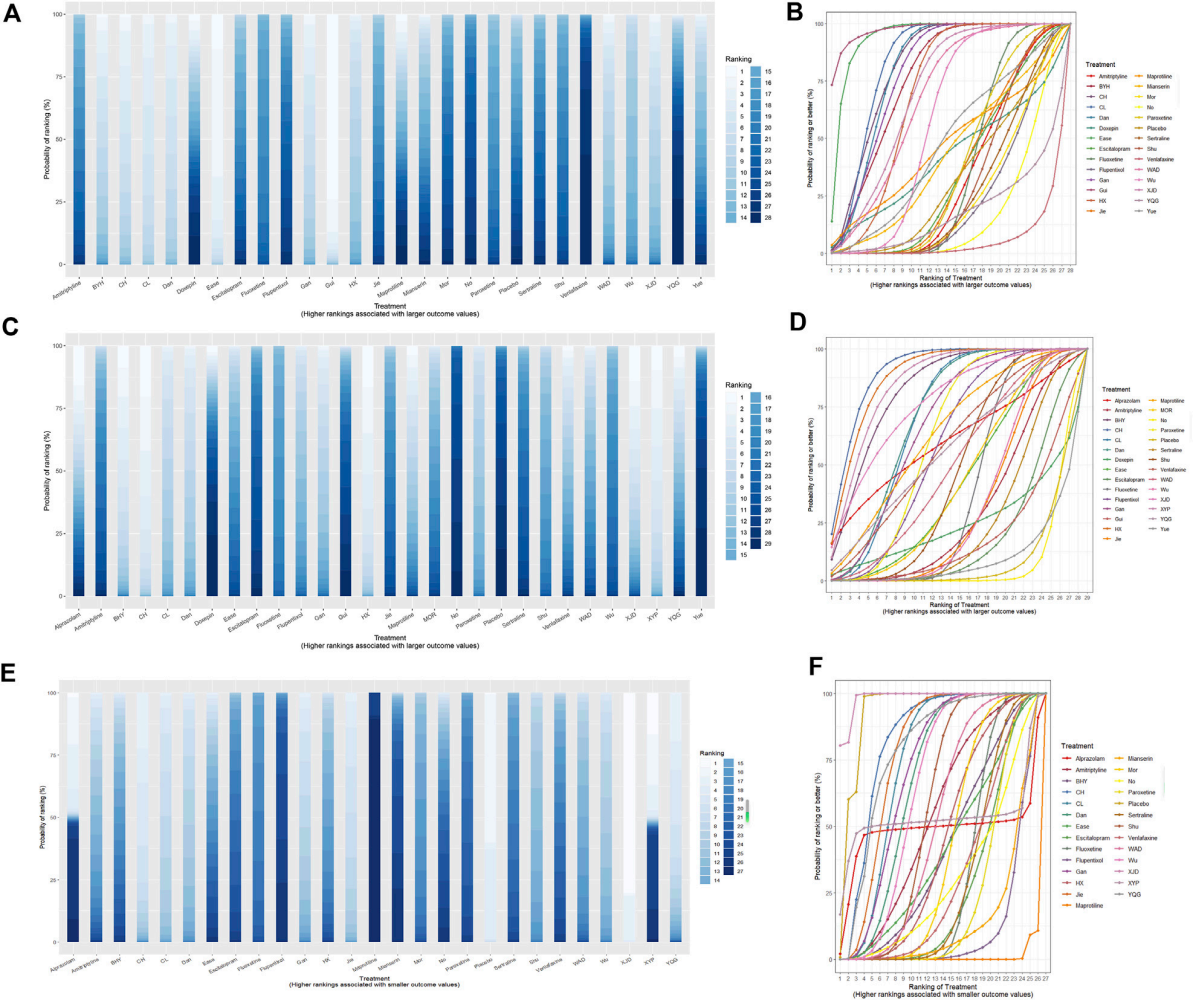
Ranking probability histogram and cumulative probability ranking chart for depression. The histogram and SUCRA charts intuitively display the sorting probability of each group in the form of histograms and curves. **(A)** Rankogram chart of response rate; **(B)** SUCRA chart of the response rate; **(C)** rankogram chart of HAMD scores; **(D)** SUCRA chart of HAMD scores; **(E)** rankogram chart of adverse effects rate; **(F)** SUCRA chart of adverse effects rate. Mor:Morinda Officinalis Oligosaccharide Capsule, Gui: Guipiwan, Jie: Jieyu Decoction, Shu: Shugan Jieyu Capsule, Wu: Wuling Capsule, Ease: Ease Pill, YQG: Yangxue Qingnao Granule, Yue: Yueju Pill, BYH: Buyang Huanwu Decoction, CL: Chaihu Jia Longgu Muli Decoction, CH: Chai Hu Shu Gan San, Dan: Danzhi-Xiaoyao-San, Gan: Gan Mai Da Zao Decoction, HX: Huoxue Soup Decoction, WAD: Wendan Anshen Decoction, XYP: Xiaoyao Powder, XJD: Xingnao Jieyu Decoction.

The results of SUCRA showed that *Guipiwan* may be the most efficacious Chinese herbal medicine to alleviate depressive symptoms but had only a small sample size. Meanwhile, the *Guipiwan* curve was higher than that of other treatments. Significantly, the total response rate of most Chinese herbal medicines was approximately superior to that of traditional antidepressants in this study. *Chai Hu Shu Gan San* was ranked best for the decrease in HAMD scores. *Xingnao Jieyu Decoction* was ranked second for reduction in HAMD scores. Moreover, *Xiaoyao Powder* was ranked best for safety in all treatments. *Maprotiline* was ranked worst for adverse effects rate with poor safety. Importantly, the safety of most Chinese herbal medicines was superior to that of traditional antidepressants in this study (Figure 9).

## Summary

This study found that specific Chinese herbal medicines, including *Guipiwan*, *Ease Pill*, *Chaihu Jia Longgu Muli decotion*, *Chai Hu Shu Gan San*, *Danzhi-Xiaoyao-San*, *Gan Mai Da Zao Decoction*, *Buyang Huanwu Decoction* and *Xingnao Jieyu Decoction*, were not only more effective than commonly used synthetic drugs (such as *Fluoxetine*, *Escitalopram*, *Amitriptyline*, *Sertraline*, *Flupentixol*, and *Venlafaxine*) but also associated with a substantially lower risk of adverse events. The findings suggest that Chinese herbal medicines could be considered as viable alternatives to synthetic antidepressants for the treatment of depression, particularly for patients who might be looking for natural remedies or those who are intolerant to the side effects of synthetic drugs. These results could inform clinical practice by expanding the range of treatment options available for depression, potentially leading to more personalized and effective treatment strategies.

## Discussion

In summary, this is the first study to systematically evaluate the safety and efficacy of 17 different Chinese herbal medicines attenuating depressive symptoms in depression patients. A Bayesian network meta-analysis was performed to explore the efficacy of single Chinese herbal medicines. The RoB2 was used to assess the methodological quality.

### Principal findings

In network diagram, *Fluoxetine*, *Shugan Jieyu Capsule*, and *Chaihu Jia Longgu Muli Decoction* were more extensively studied, followed by *Paroxetine*, *Danzhi-Xiaoyao-San*, and *Jieyu Decoction*. In heatmap, the interventions of *Buyang Huanwu Decoction*, *Chai Hu Shu Gan San*, *Chaihu Jia Longgu Muli Decoction*, *Danzhi-Xiaoyao-San*, and *Ease Pill*, *Gan Mai Da Zao Decoction* and *Guipiwan* presented more encouraging point estimates. Through direct comparison of forest map, the treatment efficacy of some Chinese herbal medicines was shown to be broadly greater than that of traditional antidepressants. According to SUCRA ranking, *Guipiwan* (SUCRA value: 96.93%) had the highest efficacy, closely followed by *Ease Pill* (SUCRA value: 93.76%), *Chaihu Jia Longgu Muli Decoction* (SUCRA value: 83.37%), *Chai Hu Shu Gan San* (SUCRA value: 81.44%), and *Danzhi-Xiaoyao-San* (SUCRA value: 80.61%). Notably, *Xiaoyao Powder* exhibited the lowest incidence of adverse events (SUCRA value: 98.23%). Moreover, commonly used synthetic drugs such as *Amitriptyline* (SUCRA value: 51.57%), *Fluoxetine* (SUCRA value: 28.48%), *Venlafaxine* (SUCRA value: 28.12%), *Escitalopram* (SUCRA value: 23.39%), *Sertraline* (SUCRA value: 26.98%), *Flupentixol* (SUCRA value: 8.51%) and *Maprotiline* (SUCRA value: 0.03%), appeared to be less effective and carried higher risks of adverse events compared to most Chinese herbal medicines. Moreover, commonly used synthetic drugs such as *Fluoxetine*, *Escitalopram*, *Amitriptyline*, *Sertraline*, *Flupentixol and Melitracen*, and *Venlafaxine*, appeared to be less effective and carried higher risks of adverse events compared to most Chinese herbal medicines.

### The mechanism of Chinese herbal medicines on depression

After thousands of years of exploration, Chinese herbal medicine has been shown advantages in the treatment of depression, such as multiple components, multitarget and strong safety, which plays a critical role in treating depression (Yeung et al., 2014b; Wang et al., 2017). The mechanisms of Chinese herbal medicines on treatment of depression are still largely unknown. The underlying pathophysiology of depression is associated with the damage of monoamine transmission systems (LeMoult and Gotlib, 2019). In fact, clinical studies have found that *Chaihu Jia Longgu Muli Decoction*, *Gan Mai Da Zao Decoction*, *Xiao Yao San* has a good antidepressant effect by preventing dopaminergic transmission in rats (Ding et al., 2021; Wan et al., 2021; Wang YT. et al., 2023). This core active ingredients of *Chaihu Jia Longgu Muli Decoction* consists of *Chaihu (Bupleurum)*, *Muli (Ostrea gigas)*, which are pivotal in treating depression (Wang Y. et al., 2023). The key active compounds in the *Gan Mai Da Zao Decoction* were identified as *Quercetin*, *Luteolin*, *Kaempfero*l, *Naringenin*, and *Isorhamnetin*, contributing significantly to its antidepressant effect (Ding et al., 2021). *Quercetin*, *Apigeni* and *Luteolin*, key components of the *Xiao Yao San*, effectively mitigate the progression of depression (Chen, 2023).

Inflammation and mitochondrial dysfunction are also associated with the pathogenesis of depression (Bansal and Kuhad, 2016; Kohler et al., 2016). In addition, *Morinda Officinalis Oligosaccharide Capsule* mitigate depression by regulating Mitofusin two protein-mediated mitophagy in rats (Yang et al., 2023). *Morinda Officinalis Oligosaccharide Capsule* mainly contains inulin-type oligosaccharides extracted from the roots of M. officinalis, which is effective in ameliorating symptoms of depressive disorder (Zhang et al., 2018). Furthermore, *Wuling Capsule* mitigate depression by regulating translocator protein-mediated mitophagy, exhibit antioxidant and anti-inflammatory effects in rats (Zheng et al., 2016). *Wuling Capsule* is mainly formulated with *Wulingshen powder*, which is a kind of fungal sclerotia of a ginseng. *Wulingshen* contains flavonoids, triterpenoids, saponins and polysaccharides, which are beneficial in improving depression (Feng et al., 2016). *Chai Hu Shu Gan San* is composed of *Chaihu (Bupleuri radix)*, *Xiangfu (Cyperus rhizome)*, and *Chuanxiong (Ligusticum chuanxiong rhizome),* which have anti-inflammatory actions and neuroprotective effects (Sun et al., 2018).

### Expectation and actual findings

The expectation of this study was Chinese herbal medicines exhibit better efficacy, and fewer side effects compared with synthetic antidepressants for the treatment of depression. It was expected to provide insights into the potential of Traditional Chinese Medicines as promising alternatives to conventional antidepressants.

The actual findings from this study are significant as they suggest that Chinese herbal medicines might be viable alternative therapies for depression, potentially offering benefits in terms of effectiveness and safety. In terms of clinical practice, these findings can inform healthcare professionals about alternative treatment options, especially for patients who may seek or prefer herbal remedies or for whom traditional antidepressants are not suitable. However, the generalizability of these results may be influenced by the study’s methodology and the specific patient populations included in the analyzed trials. Further research is needed to explore these findings in varied clinical settings and among diverse patient populations to fully ascertain the generalizability and practical application of the study’s conclusions.

### Potential confounding factors or biases

The variation in risk of bias across different studies may impact outcomes, potentially affecting the reliability of comparisons between Chinese herbal medicines and synthetic drugs. Chinese herbal medicines and synthetic drugs often differ in their mechanisms of action, side effects, and patient adherence rates. These differences could introduce confounding factors in comparative analyses. The acceptance and use of Chinese herbal medicines might be influenced by cultural beliefs and practices, which could affect patient outcomes. Geographic locations of these studies could also introduce biases, as herbal medicine practices may vary significantly across regions. Specific characteristics of patient populations in the studies, such as severity of depression, age, gender, and comorbidities, can influence the effectiveness and safety of the treatments.

### Strengths and limitations

We performed a comprehensive literature search focused on depression, addressed crucial outcomes, and rigorously assessed risk of bias at the level of evidence. The acceptability of various interventions was assessed based on criteria such as response rate, HAMD scores, and rate of adverse events on *versus* direct and indirect comparisons, thereby enhancing the persuasiveness of the evidence.

Traditional Chinese medicines are emerging as promising new drug candidates for depression treatment (Huhn, et al., 2020). This study aims to aims to determine the effectiveness, acceptability, and safety of Chinese herbal medicines in comparison with synthetic antidepressants. In addition, this study provides reference information suggesting that Chinese herbal medicines could serve as viable alternative therapies as natural remedies.

However, there are some limitations in this study. HAMD scores were used as the efficacy outcomes. Nevertheless, the data from other depression scales, such as Self-rating Depression Scale scores and Hamilton Anxiety Scale scores, were excluded due to a lack of sufficient clinical trials. These findings may lead to more complete conclusions about Chinese herbal medicines on depression. Remarkably, this study did not compare the multiple Chinese herbal medicine treatments according to the severity of depression. This review included numerous studies with small sample sizes, which limited the certainty of current evidence for the clinical use of Chinese medicines (Bian et al., 2020). Therefore, larger, more rigorous trials are necessary in the future.

## Conclusion

The study is the first to systematically assess the efficacy and safety of traditional Chinese medicines for treating depression patients using Bayesian network meta-analysis. We conclude that *Guipiwan*, *Ease Pill*, *Chaihu Jia Longgu Muli Decoction*, *Chai Hu Shu Gan San*, *Danzhi-Xiaoyao-San*, *Buyang Huanwu Decoction*, *Xiaoyao Powder*, *Huoxue Soup Decoction*, *Wendan Anshen Decoction*, *Wuling Capsule*, and *Yueju Pill* have great promise for treating depression. Further research is necessary in larger sample sizes, diverse patient populations, long-term efficacy and safety investigations comparing multiple Chinese herbal medicine treatments based on depression severity.

## Data Availability

The raw data supporting the conclusion of this article will be made available by the authors, without undue reservation.
