# Supplementary figures and images for: Chinese herbal medicines for the treatment of depression: a systematic review and network meta-analysis
Source: Front Pharmacol. 2024 Apr 3;15:1295564. doi: 10.3389/fphar.2024.1295564 (PMC11021639; doi:10.3389/fphar.2024.1295564)

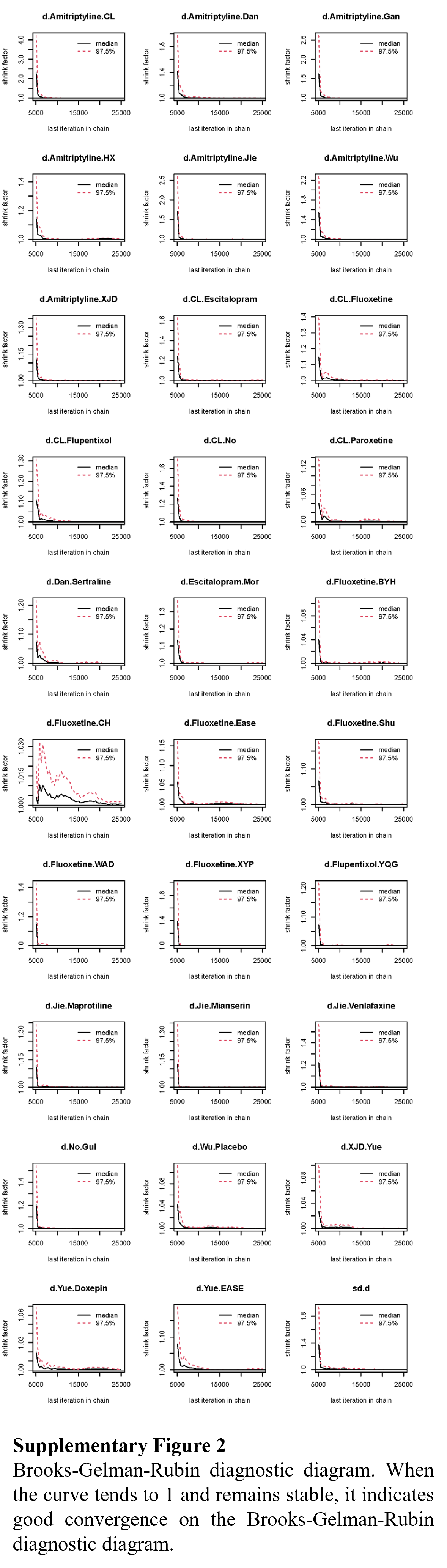

Supplement: Supplementary file 1 [file Image2.TIF]
